# A novel CRISPR/Cas9-based iduronate-2-sulfatase (IDS) knockout human neuronal cell line reveals earliest pathological changes

**DOI:** 10.1038/s41598-023-37138-5

**Published:** 2023-06-25

**Authors:** Lorenzo Badenetti, Rosa Manzoli, Marta Trevisan, Francesca D’Avanzo, Rosella Tomanin, Enrico Moro

**Affiliations:** 1 Department of Women’s and Children’s Health, University of Padova, 35128 Padova, Italy; 2 Istituto di Ricerca Pediatrica “Città Della Speranza”, 35127 Padova, Italy; 3 Department of Molecular Medicine, University of Padova, 35121 Padova, Italy; 4 Department of Biology, University of Padova, Via Ugo Bassi 58/B, 35121 Padova, Italy

**Keywords:** Cell biology, Diseases

## Abstract

Multiple complex intracellular cascades contributing to Hunter syndrome (mucopolysaccharidosis type II) pathogenesis have been recognized and documented in the past years. However, the hierarchy of early cellular abnormalities leading to irreversible neuronal damage is far from being completely understood. To tackle this issue, we have generated two novel iduronate-2-sulfatase (IDS) loss of function human neuronal cell lines by means of genome editing. We show that both neuronal cell lines exhibit no enzymatic activity and increased GAG storage despite a completely different genotype. At a cellular level, they display reduced differentiation, significantly decreased LAMP1 and RAB7 protein levels, impaired lysosomal acidification and increased lipid storage. Moreover, one of the two clones is characterized by a marked decrease of the autophagic marker p62, while none of the two mutants exhibit marked oxidative stress and mitochondrial morphological changes. Based on our preliminary findings, we hypothesize that neuronal differentiation might be significantly affected by IDS functional impairment.

## Introduction

Among different mucopolysaccharidoses (MPSs), mucopolysaccharidosis type II (MPS II), known as Hunter syndrome (OMIM 309,900), is the only X-linked trait characterized by heparan-sulfate (HS) and dermatan-sulfate (DS) storage with a high HS/DS ratio^1^. The disease is caused by a deficit in the activity of the lysosomal enzyme iduronate-2-sulfatase (IDS) due to mutations affecting the *IDS* gene. The gene localizes in the chromosomal region Xq28, spans 44 kb and it is composed of nine exons. A pseudogene, called *IDSP1*, containing sequences homologous to *IDS* exons 2, 3 and introns 2, 3 and 7, is located 3.9 kb far from *IDS*, telomeric to the gene and in the opposite orientation^2^. This favors the phenomena of homologous recombination between gene and pseudogene, further increasing the already elevated number of genomic variants characterizing *IDS*. Hunter syndrome exhibits a relatively early onset, with almost two thirds of the patients developing central nervous system (CNS) manifestations at 2–4 years of age. Severely affected patients often experience progressive cognitive deterioration with behavioral problems and neurological abnormalities that can lead to their premature death^3^. Among different therapeutic approaches, the gold standard treatment of MPS II patients, i.e. Enzyme Replacement Therapy (ERT), relies on the administration of a recombinant enzyme formulation. However, ERT cannot alleviate neurological symptoms since the presently used formulation of the recombinant enzyme is unable to cross the blood–brain barrier (BBB). Recently, a novel chimeric recombinant enzyme (*pabinafusp alfa*) bypassing the BBB has received growing attention. Phase II and III clinical trials in Brasil and Japan with *pabinafusp alfa* on affected patients have shown important changes in the CNS disease trajectories, making this novel ERT formulation quite promising^4,5^. Alternative therapeutic approaches consist of Hematopoietic Stem Cell transplantation (HSCT) or Hematopoietic Stem Cell transplantation Gene Therapy (HSC-GT). A previous retrospective study performed by Kubaski and colleagues showed significant improvements in HSCT-treated MPS II patients when compared to the ERT-treated ones^6^. Regarding the HSC-GT, a recent non-randomized phase I/II clinical trial (AVR-RD-05, NCT05665166), based on preclinical studies on a MPS II mouse model^7,8^, has been approved in UK and planned to begin in early 2023. Despite the growing development of potential beneficial treatments, the lack of a deep knowledge of disease pathogenesis, together with limitations related to long-term effects and relatively high costs for each HSCT/HSC-GT treated patient, support the need of increasing the repertoire of preclinical models. Several experimental in vitro and in vivo MPS II models have been generated in the past years to address the identification of pathogenic aspects of the disease^9–13^. Regarding in vitro models, molecular changes often introduced by immortalization of neuronal cell lines have prompted the alternative use of human induced pluripotent stem cells (iPSCs) derived from patient fibroblasts, as standard experimental model. In the context of MPS II, different research groups previously attempted to recapitulate most primary and secondary cellular manifestations, including the lipid accumulation and defects in the autophagic machinery^11,12,14^. However, the use of reprogrammed iPSCs suffers from the primary issue that a control with a genetically matching background and common origin (isogenic) is often missing, making all experimental observations potentially incorrect and misleading. Moreover, the generation of iPSCs from patients’ fibroblasts and their reprogramming into neurons is often time consuming and quite expensive. To circumvent these problems, we here present the generation and characterization of a novel human MPS II neuronal model, which may be a valuable alternative to investigate the pathogenic mechanisms of the disease. We provide a preliminary description of two complete IDS loss of function clones, exhibiting common and consistent primary defects in their differentiation potential, as well as in the endolysosomal compartment. We also show that differentiation and endolysosomal defects concur with primary lipids storage, while we were not able to detect gross mitochondrial abnormalities and oxidative stress. Our results support the evidence that IDS loss of function is extremely relevant for the early differentiation of neuronal precursors, as well as for their intracellular vesicle trafficking.

## Results

### Generation of human IDS deficient neuronal cells

**Figure 1:**
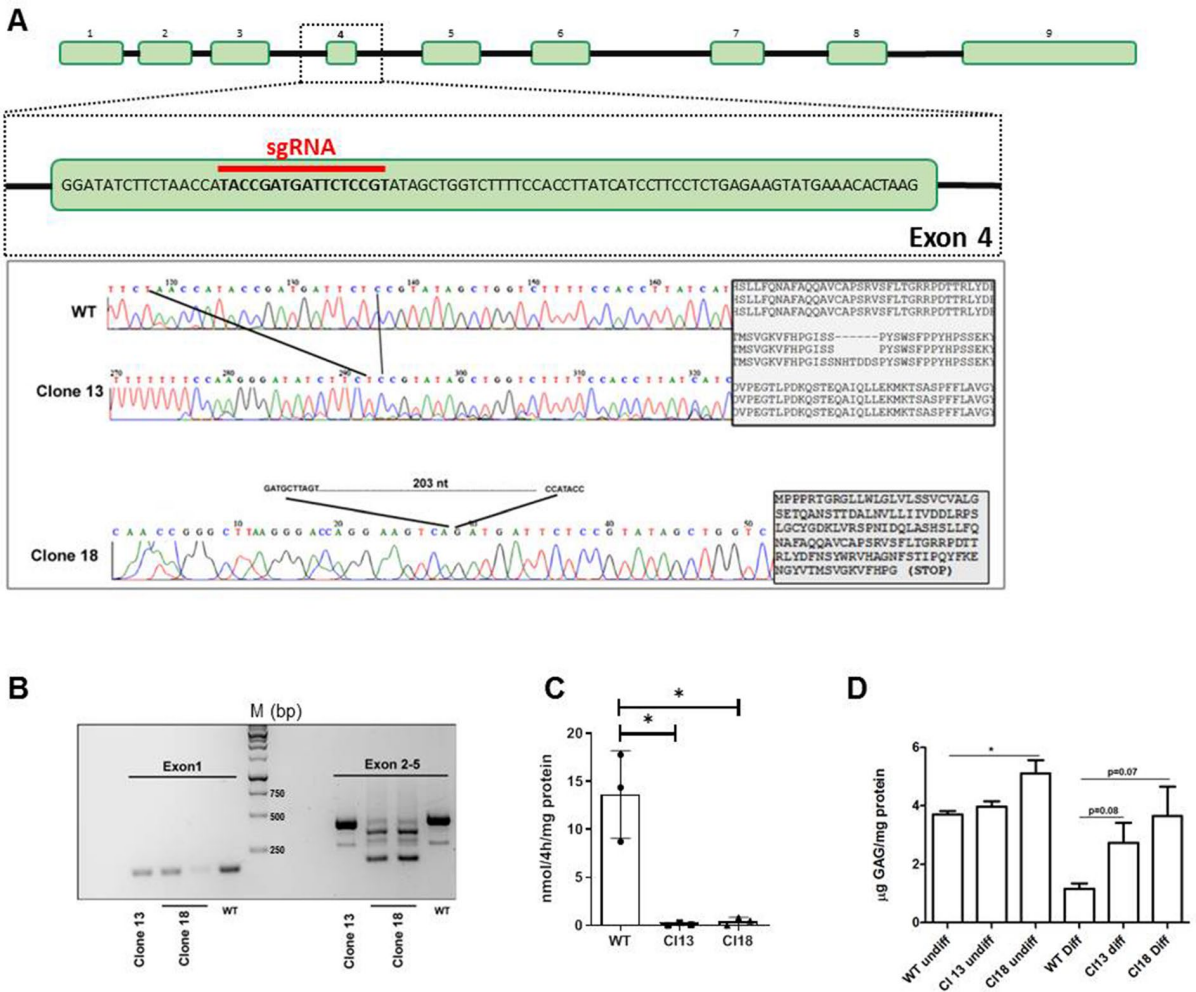
Generation and validation of human MPS II neuronal cells. (**A**) (top) Schematic representation of the *IDS* coding region (exons and introns) with the sgRNA targeting sequence at exon 4. (bottom) Chromatograms of the DNA sequences from wild type and *IDS* mutant clones. The 18 nucleotide sequence of exon 4 missing in clone 13 is shown between the two dark lines. On the right the alignment of the aminoacidic sequence between clone 13 and wild-type control cells depicts the missing aminoacidic stretch (NHTDDS) detected in mutant cells. On the bottom the missing 203 nucleotide sequence and the predicted truncated IDS protein of clone 18 is reported. (**B**) Agarose gel electrophoresis of *IDS* exon 1 and exon 2–5 amplicons from wild-type control and *IDS* mutants are shown. (**C**) Iduronate-2-sulfatase enzymatic activity assay performed in undifferentiated control and mutant cells. Data are expressed as the mean ± SD of three independent sub-clones (*p < 0.05 t-test). (**D**) GAG assay performed on undifferentiated and differentiated (d5) control and mutant cells. Data displayed are means ± SD of two independent differentiation processes.

To create an in vitro experimental system amenable for rapid and reproducible phenotypic analyses of human IDS-deficient neuronal cells, we took advantage of the Lund Human Mesencephalic (LUHMES) cell line, which derives from human embryonic mesencephalic precursors and can be rapidly differentiated into dopaminergic neurons^15^. Using a single guide RNA (sgRNA) designed against *IDS* exon 4 (Fig. 1A), we were able to mutagenize LUHMES cells by CRISPR/Cas9. From twenty isolated single mutagenized cell clones by Sanger sequencing we could measure an overall 50% indel efficiency in the bulk cell population. However, more than 20% detected indels were carrier of a 3-aminoacid deletion. Of the remaining mutant clones two were selected for downstream analysis given their profile by PCR (data not shown) and RT-PCR analyses (Fig. 1B). Indeed, by Sanger resequencing we confirmed that the suspected indels were de facto* IDS* mutants. The first one, from herein referred as clone 13, was characterized by a 18 nucleotide (i.e. six aminoacid) deletion in exon 4 (from aminoacid 144 to 149 from the first methionine), leading to the loss of one of the known IDS glycosylation sites (Asparagine 144)^16^. The second clone, from herein referred as clone 18, was carrying a 203 nucleotide deletion including twenty nucleotides of exon 4 which, according to the frameshift generated, was expected to produce a truncated protein. We first performed a preliminary Western Blot on pooled protein lysates from undifferentiated control and mutated cells and we could not detect at least for clone 18 the band corresponding to the deglycosylated form of the enzyme (Fig. S1). We next performed multiple enzymatic assays from independent sub-clones of the two mutants and control cells and consistently found that both mutants were characterized by the complete lack of enzymatic activity (Fig. 1C). To further confirm the correct *IDS* targeting, we carried out the analysis of total glycosaminoglycans (GAGs) levels in undifferentiated and differentiated cells and we found that differentiated mutant clones exhibited increased GAGs levels (Fig. 1D). Therefore, we could conclude that our genome editing protocol was successfully able to target the *IDS* genomic sequence, yielding to two complete loss of function mutations.

### IDS mutant neuronal cells exhibit impaired differentiation into dopaminergic neurons at earliest stages

**Figure 2:**
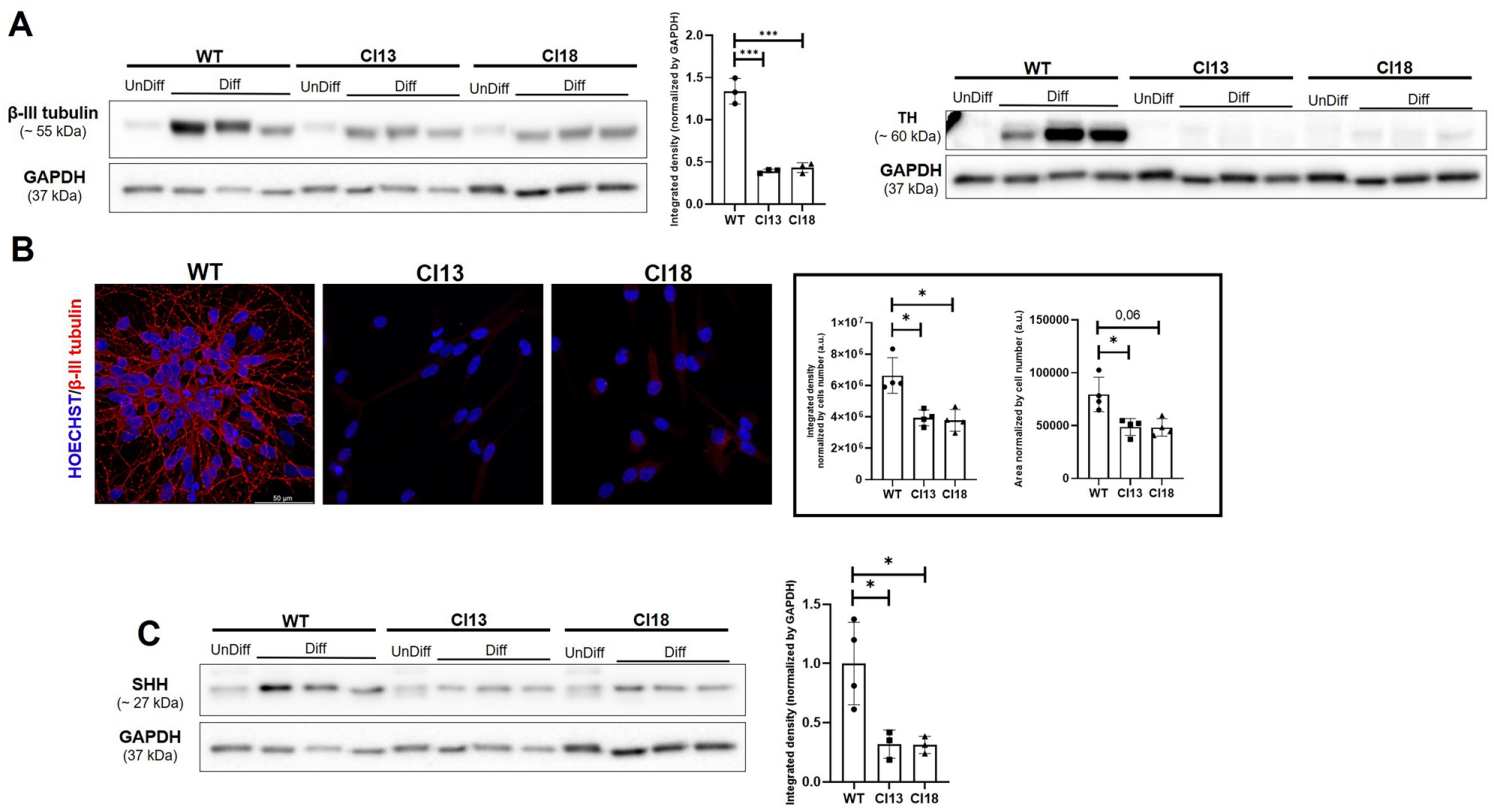
Reduced differentiation of MPS II neuronal cells. (**A**) Representative Western Blot performed on undifferentiated (unDiff) and differentiated (Diff) control and MPS II neuronal cells, showing significantly decreased β -III tubulin and TH protein levels in mutant cells. The bar-graph in the middle depicts the densitometric analysis of the β-III tubulin-related Western Blot performed on samples obtained from three differentiation processes at d5 of three independent sub-clones. For the TH marker mutant clones showed an undetectable band, which could not be evaluated by densitometric analysis. (**B**) Representative immunofluorescence for β-III tubulin in differentiated control and mutant cells. The bar graphs depicts the integrated density and the area of fluorescence measured in at least 100 hundred cells per condition (N = 4 replicates obtained by differentiation at d5 of four independent sub-clones) (**C**) Representative Western Blot performed on control and *IDS* mutant clones, showing the significantly reduced SHH protein levels detected in mutant cells. Data are expressed as the mean ± SD of three replicates obtained by the differentiation at d5 of three independent sub-clones (*p < 0.05; ***p < 0.001; t-test).

Since the LUHMES cell line can be rapidly differentiated into mature dopaminergic neurons^15^, we sought to investigate whether the lack of IDS enzymatic activity could affect the differentiation potential of the obtained mutant clones. To this purpose, we first assessed by Western Blots the amount of β-III tubulin (earliest neuronal marker) and tyrosine hydroxylase (TH) protein levels in protein extracts from multiple independent differentiation tests. As shown in Fig. 2A, we could detect significantly decreased β-III tubulin levels (nearly three-fold), and almost no TH protein levels in both clones. A resembling dysregulation of β-III tubulin in mutant clones was also detectable by immunostaining (Fig. 2B). To further verify whether the decrease of TH protein levels was paralleled by reduced *TH* gene transcription, we carried out RQ-PCR tests on pooled RNAs from differentiated control and mutant cells and we found a significant decrease of *TH* mRNAs for both clones (Fig. S2). Since the ciliary canonical Shh signaling is actively implicated in the LUHMES differentiation^17^, we next performed Western Blot analyses for the SHH ligand in the same protein extracts and we consistently found significantly decreased SHH protein levels in mutant clones when compared to those of unmutated control (Fig. 2C). Therefore, we could conclude that the loss of IDS function in neuronal cells negatively impact on SHH ligand levels and neuronal differentiation.

### The autophagosomal and endosomal-lysosomal systems of neuronal cells are negatively affected by IDS loss of function

**Figure 3:**
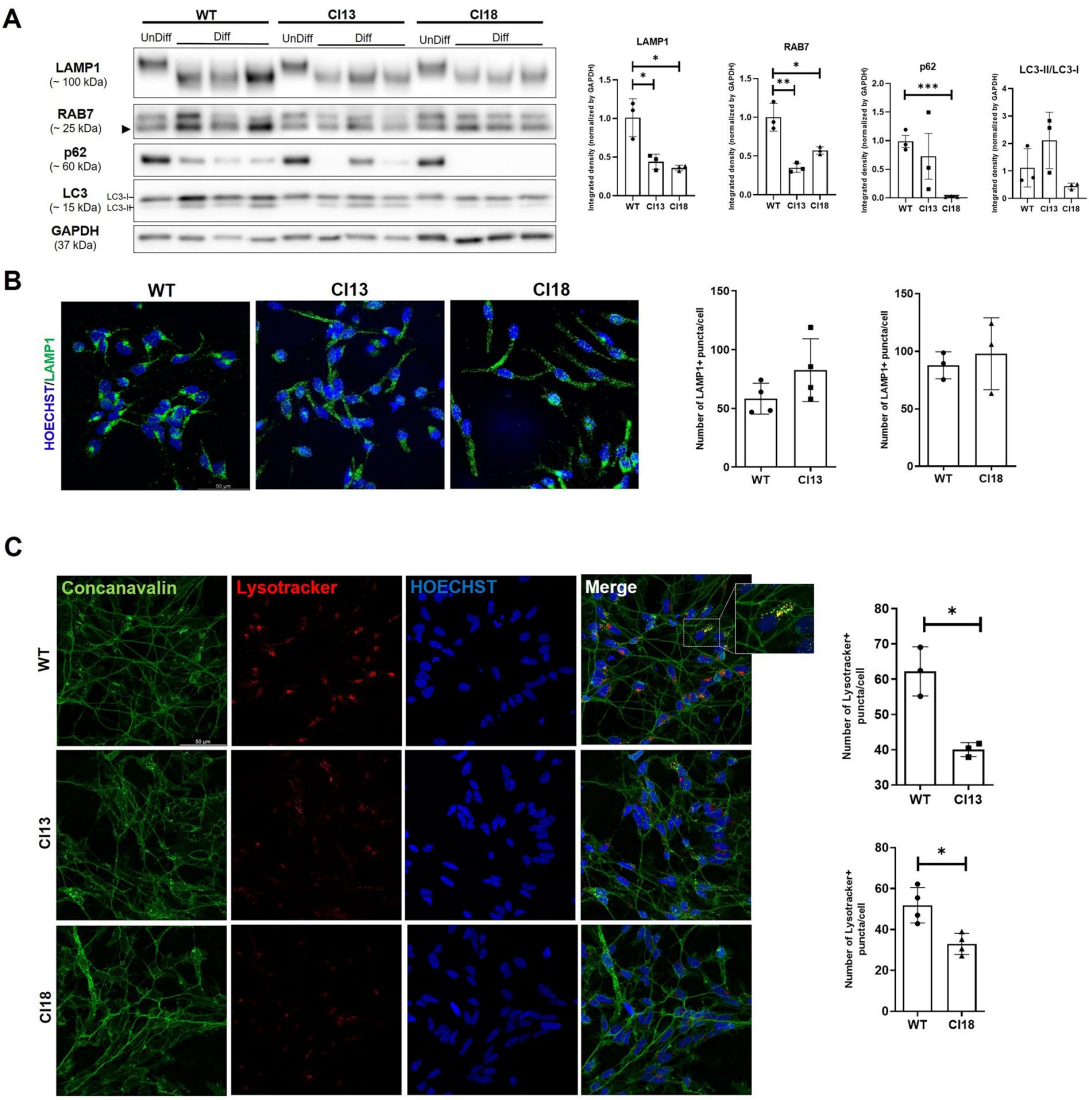
Decreased endolysosomal markers levels and lysosomal acidification defects in MPS II neuronal cells. (**A**) Representative Western Blots for p62, LC3 (LC3-I/LC3-II), LAMP1 and RAB7 in undifferentiated (UnDiff) and differentiated (Diff) control (WT) and mutant (Cl13 and Cl18) cells at d5. Data are expressed as the mean ± SD of three replicates obtained by three independent sub-clones differentiated at d5. Note that, while LAMP1 and RAB7 protein levels are significantly reduced in differentiated mutant cells of both clones, decreased p62 levels are detected only in clone 18 when compared to control. (**B**) Representative immunofluorescence for LAMP1 in control and mutant cells show a “scattered” localization pattern in mutant cells, when compared to the control, where LAMP1-positive puncta are preferentially located in axonal hillocks. On the right, bar graphs depict no differences in the number of puncta measured on whole Z-stacks from several cells (at least 700 cells were analyzed per condition in replicates obtained by three differentiation processes at d5 of three independent sub-clones). (**C**) Representative double Concanavalin/Lysotracker staining in differentiated control and mutant cells and ImageJ-based quantifications (bar graphs on the right) indicate significantly reduced Lysotracker staining occurring in mutant cells. A magnification of control cells co-stained for Lysotracker/Concanavalin depicts the preferential localization of acidified lysosomes on the axonal hillock. Data are expressed as the mean ± SD of three replicates obtained by the differentiation at d5 of three independent sub-clones (400 cells were analyzed per condition of each replicate) (*p < 0.05; **p < 0.005; ***p < 0.001; t-test).

Considering the important role of the autophagosomal and endosomal-lysosomal systems in neuronal homeostasis^18^ and given previous findings in different experimental models^19^, we next explored whether the loss of IDS function could affect the intracellular degradative system. To this purpose, we tested our mutants for a set of known markers for autophagy and late endosome-lysosomes. When assayed for the cargo adaptor p62 and the autophagosomal markers Microtubule-Associated Protein 1 Light Chain 3 (LC3-II/LC3-I), we found that only clone 18 was characterized by a significant decrease of p62 protein levels. Indeed, none of the two clones showed significantly changed LC3-II/LC3-I protein levels (Fig. 3A). Regarding the endosomal-lysosomal system, we next performed Western blot analyses for the late endosomal marker Ras-associated binding protein 7 (RAB7) and the endolysosomal marker Lysosomal Associated Membrane Protein 1 (LAMP1) on protein lysates from *IDS* mutant and control cells. For both markers we found significantly decreased protein levels in mutant cell extracts, when compared to those of the unmutated control (Fig. 3A). We also verified these findings by performing several immunofluorescence analyses for LAMP1 on differentiated mutant and control cells. As shown in Fig. 3B, we noticed that, while in some control cells most LAMP1 positive puncta were located in the axonal hillock, in mutant cells LAMP1-positive puncta were scattered in the soma and along enlarged axonal projections. In most cases, however, we could not detect significantly reduced LAMP1 positive puncta in mutant cells when compared to the unmutated control (see bar graphs, Fig. 3B). To further assess whether the loss of IDS activity detected in both mutants could impact on lysosomal acidification, we performed the Lysotracker staining on control and mutant cells at the same stage of differentiation (day 5 of differentiation, d5) and compared by confocal imaging their profile. In all independent assays, we consistently detected in mutant cells a significantly decreased number of Lysotracker puncta when compared to unmutated cells (Fig. 3C). Therefore, according to these results, we could conclude that the endolysosomal compartment and lysosomal acidification are negatively affected in both mutant clones. However, for only clone 18, the autophagic marker p62 was significantly reduced by IDS loss of function.

### No evident oxidative stress and mitochondrial defects are detected in IDS mutant neuronal cells

**Figure 4:**
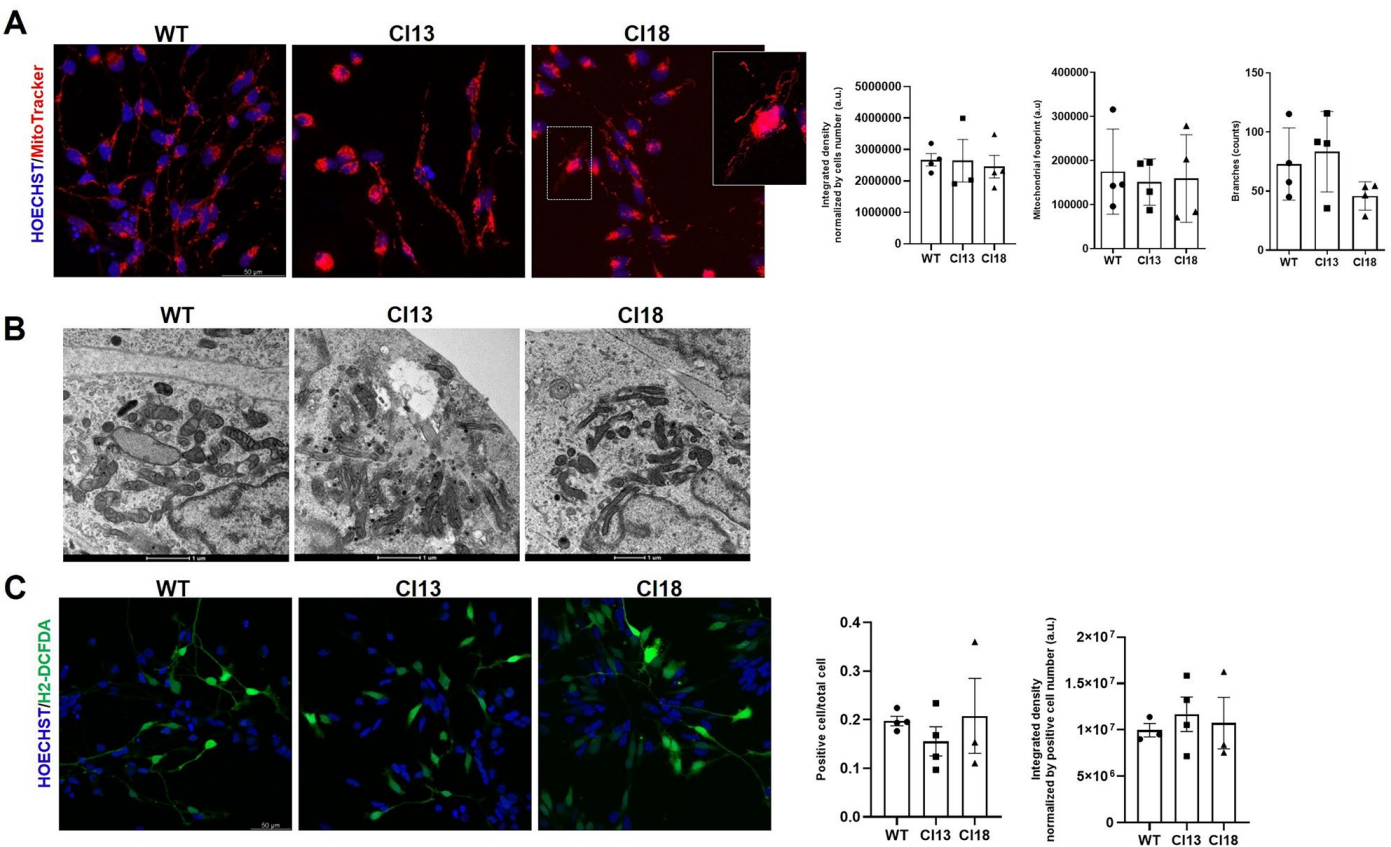
Lack of evident mitochondrial abnormalities and oxidative stress in early differentiated MPS II neurons. (**A**) Representative Mitotracker staining and ImageJ-based quantifications of differentiated control and mutant cells showing no gross abnormalities, except the presence of filamentous mitochondria (see magnification of clone 18 panel). Data are expressed as the mean ± SD of three replicates obtained by the differentiation at d5 of three independent sub-clones (300 cells were analyzed per condition of each replicate). (**B**) Representative T.E.M. images of differentiated control and mutant neuronal cells. No gross mitochondrial abnormalities are detectable between control and mutant cells. (**C**) Representative H2-DCFDA staining and ImageJ-based quantifications of differentiated control and mutant cells showing no significant ROS accumulation in mutant cells. Data are expressed as the mean ± SD of three replicates obtained by the differentiation at d5 of three independent sub-clones (400 cells were analyzed per condition of each replicate).

Since the aberrant autophagic and endolysosomal machinery may prevent the clearance of defective mitochondria^20^, we sought to explore whether the mitochondrial compartment could be affected by the complete loss of IDS function in our mutant neuronal cell clones. Towards this aim, we first stained with the Mitotracker dye both clones and unmutated control after differentiation and analyzed their mitochondrial network using the MiNA platform (see “Methods”). As shown in Fig. 4A, while we noticed that in both mutant clones individual cells were exhibiting long filamentous mitochondria patterning, we did not find significant differences in the mitochondrial network morphology between unmutated and mutated cells, nor differences in the amount of Mitotracker-stained areas. We next examined by transmission electron microscopy (T.E.M.) the mitochondrial morphology of both differentiated mutant clones, but we could not detect gross morphological changes related to the mitochondrial compartment when compared to that of wild type cells (Fig. 4B). To further verify whether oxidative stress could affect the differentiation status of mutant cells, we stained them with the fluorogenic probe dichlorodihydrofluorescein diacetate (H2-DCFDA), but we could not detect significant differences when compared to unmutated cells (Fig. 4C). Taken together, these results suggest that the loss of IDS function in our mutant clones does not appear to significantly affect the mitochondrial compartment, nor it appears to be associated with increased oxidative stress during early stages of differentiation.

### IDS loss of function neuronal cells exhibit secondary lipids storage at early differentiation stages

**Figure 5:**
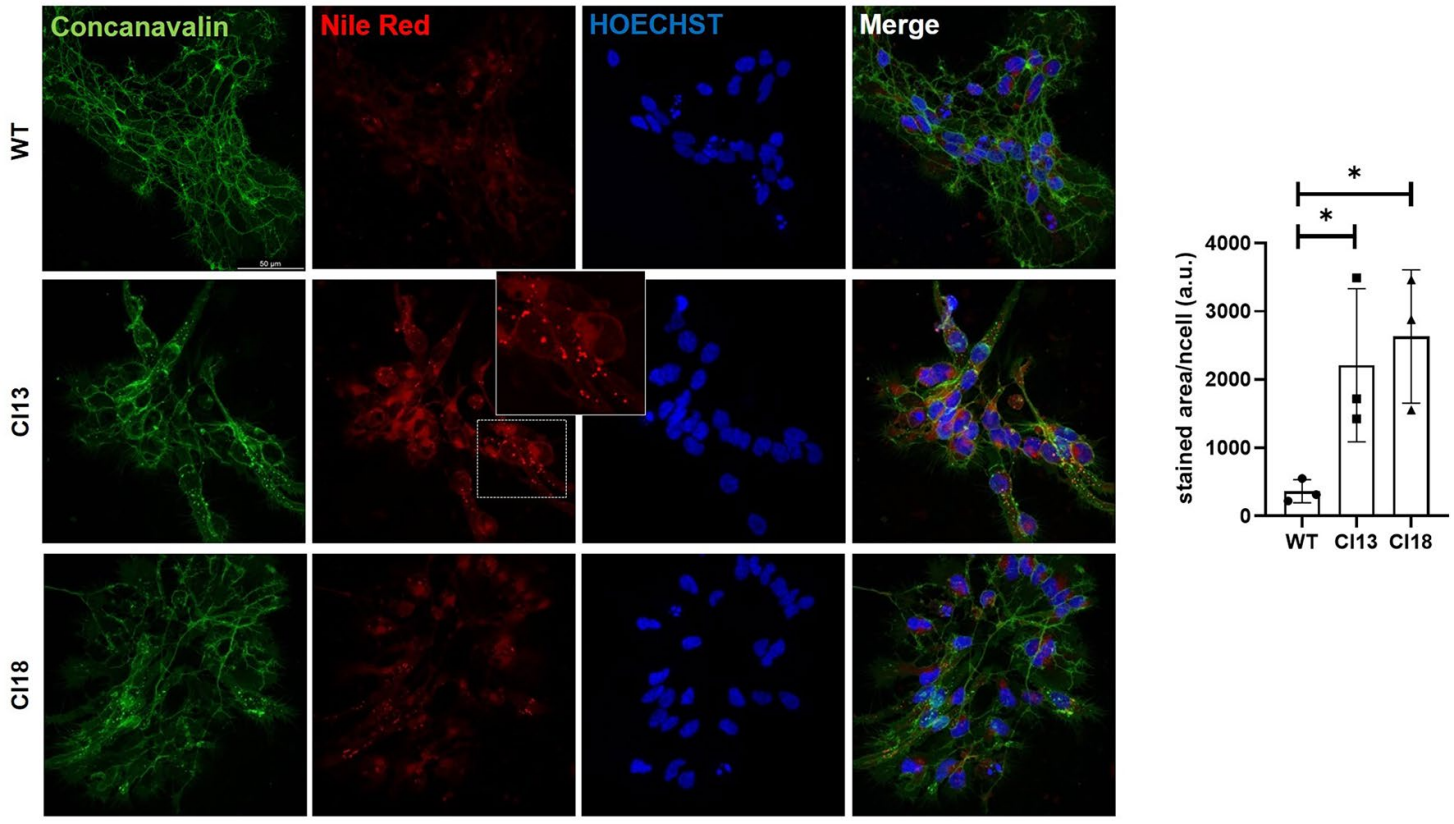
Increased lipid storage is detected in early differentiated MPS II neuronal cells. Representative double Concanavalin/Nile Red staining in differentiated (d5) control and mutated neurons. A magnification of Nile red-stained puncta observed in mutant cells is depicted in the panel of clone 13. The bar graph on the right depicts the area of positive Nile Red stained dots normalized per cell. Data are expressed as the mean ± SD of three technical replicates obtained from a single differentiation process (about 150 cells were analyzed per each technical replicate).

A characteristic hallmark of mucopolysaccharidoses is the progressive secondary storage of substrates, including cholesterol and sphingolipids^21^. To address whether during early differentiation IDS mutants may store lipids and cholesterol, we performed Nile Red and TopFluor®Cholesterol (Top-Chol) staining on control and mutant cells at d5 (Fig. 5 and Fig. S3). While we found that both mutants were characterized by increased lipids storage (Fig. 5), none of them exhibited increased lysosomal cholesterol, when compared to unmutated control neurons (Fig. S3). Therefore we could conclude that, while cholesterol does not appear to significantly increase within cells during early differentiation, lipids are already accumulating in *IDS* mutant neurons.

## Discussion

Hunter syndrome is a rare lysosomal disorder whose neurological manifestations in severely affected patients arise during early childhood, leading to progressive cognitive deterioration and behavioral abnormalities^3^. A longstanding question emerged in the past years is how the loss of IDS activity impacts the neuronal compartment and which primary events may take place within neurons, as a consequence of impaired lysosomal activity. To address this issue, several in vitro and in vivo experimental models have been generated and characterized to define the spatio-temporal dynamics of tissue-specific alterations^22^. These studies have led to the progressively growing awareness that many concurrent cellular defects occur downstream of lysosomal dysfunction before the onset of massive lysosomal substrate storage^23^. Among them, using a zebrafish MPS II model, we previously documented the perturbation of distinct cell signaling pathways, particularly Fgf, Shh and Wnt at the cardiac and bone levels^10,24^. All these aberrant phenomena were already evident at early developmental stages and preceded the onset of tissue specific defects. Given the recently reported implication of the dopaminergic neurons in the behavioral abnormalities of MPS II and MPS IIIA mice^25^, we sought to verify in this work whether IDS loss of function may impact the neuronal differentiation potential using a novel in vitro human neuronal model for MPS II (CRISPR/Cas9 mutated LUHMES cell line). The advantage of this model stands on its relatively easy protocol of differentiation^15^. Additionally, it circumvents the potential issue of finding syngeneic unmutated controls when using iPSC-derived neuronal cells as MPS II models. Indeed, we were able to retrieve two independent IDS loss of function clones: the first one (clone 13), harboring a deletion of six aminoacids comprising the N144 glycosylation site, did not exhibit IDS enzymatic activity in agreement with the study of Millat and colleagues^16^. For the second mutant clone (clone 18), also displaying no enzymatic activity, we could not find a similar molecular defect in the HGMD database (https://www.hgmd.cf.ac.uk), although some of the mutations reported in the same database similarly involved exon 4.

One of the major findings of this investigation was that full IDS loss of function negatively impact on the neuronal differentiation, particularly into dopaminergic neurons. Analyses of neuronal differentiation have been previously made in MPS II and MPS IIIA mice^26,27^. Notably, De Risi and colleagues, using primary cultures from mesencephalic neurons of MPS IIIA mice showed progressively decreased TH + -cell density, which Authors pointed as a result of increased cell death^25^. In our mutant cellular model, we measured significantly decreased β–III tubulin levels and almost undetectable TH protein levels at 5 days post-differentiation, when no marked differences were measured between wild type and mutant clones viability (0% trypan blue positive wild type cells vs 0% clone 13; 0% trypan blue positive wild type vs 4% clone 18). In agreement with our observations, neuronal differentiation defects have been also detected in a human iPSC-based model of metachromatic leukodystrophy (MLD)^26^ and MPS IIIA^27^. A second main observation in our MPS II neuronal model was the detection of reduced levels of some endolysosomal proteins (RAB7 and LAMP1) and decreased lysosomal acidification. In contrast with our results, recently characterized iPSCs-derived MPS II neural stem cells (NSCs) have been shown to exhibit increased LAMP1 immunostaining areas^14^. However, we noticed that Authors only performed the integrated intensity analyses of immunostained cells. We, indeed, found that after many independent differentiation protocols, LAMP1 protein levels were consistently decreased in both mutant clones by Western Blot, while no significant differences were detected by the integrated density analysis, as well as by counting the number of LAMP1-positive spots in immunostained cells. We, therefore, assume that the limited sensitiveness of integrated density analysis may justify this discrepancy. On the other hand, since LAMP1 is also partially considered a late endosomal marker^28^, our observation perfectly agrees with the detection of reduced RAB7 protein levels in the same IDS mutants. We also observed that in mutant cells LAMP1-positive puncta were scattered in the cytoplasm and less concentrated in the axonal hillock, as the ones in control cells (Fig. 3B). This different LAMP1 localization patterning could be ascribed to an abnormal morphology displayed by mutant cells, which failed to form dense neuronal networks in differentiating conditions. Regarding the decreased lysosomal acidification exhibited by our MPS II mutants, we searched in literature and found no documented analyses of Lysotracker staining in human differentiated MPS II neurons. While for some lysosomal disorders (NPC, MPS IIIB) increased lysosomal acidification has been demonstrated, lysosomal alkalinization was consistently documented in neuronal ceroid lipofuscinoses (NCLs)^29^ and Krabbe disease^30^. Notably, lysosomal alkalinization has been pointed out as detrimental also in other neurodegenerative diseases^31,32^. While for clone 13 we were not able to detect autophagic markers changes, we documented significantly decreased p62 protein levels in clone 18. Reduced p62 protein levels have been previously reported in Gaucher and Fabry disease peripheral blood mononuclear cells (PBMCs)^33^. We hypothesize that the degree and kinetics of autophagic markers changes, preluding to an autophagic defect, might be affected by the type of mutation, although the molecular mechanism remains purely speculative. Finally, in agreement with recent observations^14^, we found increased lipids storage in mutant clones, while we did not observe lysosomal cholesterol storage few days after differentiation. The red emission spectrum of Nile Red, through which we analyzed our cells, is more restricted to polar lipids, including unsaturated glycerophospholipids and sphingolipids^34^. Therefore, the undetected cholesterol storage well fits with previous observations made in the cerebrospinal fluid (CSF) of MPS II patients, where the most abundant lysosomal lipids detected by liquid chromatography-mass spectrometry (LC–MS) assays were sphingolipid species^35^. In conclusion, in this work we provide the description of a novel tool which allows to investigate early primary pathogenic mechanisms in the MPS II neuronal population. Moreover, the novel MPS II neuronal cell lines we generated may be exploited to rapidly test novel small molecules to be used for targeting MPS II-related aberrant pathogenic mechanisms.

## Methods

### Cell culture and differentiation

Lund Human Mesencephalic (LUHMES) neuronal cells were purchased from ATCC (CRL 2927) and cultured according to previous reports^15^ and manufacturer’s instructions. Briefly, all plastic culture plates were pre-coated with 50 μg/ml poly-L-ornithine (Merck, Italy) overnight, washed twice with sterilized water at the end of incubation and then treated with either 1μg/mL fibronectin (Merck, Italy) alone for three hours at 37 °C, or in combination with an overnight incubation with 10 μg/mL laminin (Glpbio, USA), as recently suggested^36^. Cells were maintained in T-75 culture flasks (Sarstedt, Italy) at 37 °C with a humified atmosphere of 95% air and 5% CO_2_ using the Advanced Dulbecco’s modified Eagle’s medium (DMEM/F12), supplemented with N-2 supplement (Thermofisher, Italy), 2 mM Glutamine (Thermofisher, Italy) and 40 ng/mL recombinant basic fibroblast growth factor (bFGF) (Thermofisher, Italy). During their propagation, culture medium was changed every other day and cells were subcultured at 1:4 ratios by standard 0.025% Trypsin/0.1 g/L EDTA-based dissociation and seeding in fresh medium. The differentiation protocol followed the optimized method suggested by Harishandra and colleagues^15^, in which the differentiation cocktail included Advanced DMEM/F12, N2-supplement, 2 mM L-glutamine, 1 mM dibutyryl cAMP, 1 μg/mL tetracycline, 20 ng/mL recombinant human GDNF, 20 ng/mL recombinant human BDNF , 10 ng/mL human recombinant LIF (all purchased from Thermofisher, Italy), 0.2 mM ascorbic acid (Merck, Italy), 20 ng/mL TGF β-III (StemCell Technologies, US). For all experiments cells at early passages were plated in 24-well plates and differentiation medium was replaced every day up to 5 days of differentiation. For each condition, the differentiation procedure was repeated many times, collecting cells from multiple wells at the end of each individual differentiation protocol. Viability was assessed by the Trypan blue method, incubating differentiated cells grown on coverslips for 2 min with a 0.2% Trypan Blue solution (Thermofisher, Italy) and after post-fixing with 4% paraformaldehyde (PFA) for 20 min coverslips were mounted and visualized under a light microscope.

### Generation of IDS mutant LUHMES cells

To generate a loss of function mutant clone, a custom-designed Alt-R CRISPR-Cas9 sgRNA targeting the 5’end of human IDS coding sequence was purchased from IDT (Leuven, Belgium). Using the CRISPR-Cas9 guide RNA design checker software provided by the company all off-target regions predicted to be recognized by the sgRNA showed a score compatible with negligible potential editing (Supplementary Table S1). Undifferentiated LUHMES cells were trypsinized with trypsin/EDTA 0.025% (Thermofisher, Italy), counted and diluted with phosphate buffered solution (PBS). One million cells were washed with PBS and re-suspended in 100 µl of Amaxa P3 solution (Lonza, Basel, Switzerland) together with a mixture of sgRNAs/Cas9 (120 pmol and 104 pmol, respectively). Cells were then electroporated with a 4D Nucleofector device (Lonza) using program CA-137 and allowed to immediately recover for 5 min at 37 °C, after adding 500 µl of medium supplemented with N2, glutamine and FGF. Upon recovery after nucleofection, transfected cells were seeded on flasks previously coated with 50 µg/mL poly-L-ornithine and then with 1 µg/mL human fibronectin. Bulk mutagenized cells were subjected to the T7 endonuclease I (T7EI) mismatch cleavage assay (IDT, Belgium) to detect potential indels, following manufacturer’s protocol. Single-cell clones were obtained by serially diluting putative mutant cells and seeding each single cell in 96-well culture plates (Sarstedt, Italy). Isolated clones were identified and picked when each colony began to expand, forming a sphere. Genomic DNA was extracted from each isolated clone by treatment with SDS buffer, phenol–chloroform extraction, precipitation in absolute ethanol and resuspension in DNAase free water. We next verified by Sanger sequencing each identified mutant clone and proceeded with downstream analyses. The sgRNA sequence and primers used for Sanger sequencing are listed in Supplementary Table S2A.

### RNA extraction and RT-PCR

Control and mutated cells were homogenized in Trizol reagent (Thermofisher, Italy) and total RNA was isolated using the standard chloroform-ethanol extraction procedure, according to manufacturer’s instructions. Total RNAs were resuspended in 20 µl of RNAse free water and then quantified by Nanodrop 1000 spectrofotometer. Two micrograms of purified RNA for each condition was reverse transcribed using a SuperScript III Reverse Transcriptase (Thermofisher, Italy), according to standard procedures. cDNAs were next subjected to PCR using oligos against a region spanning the human IDS exon 1 or from exon 2 to exon 5 (see Supplementary Table S2A). PCR products were finally run onto an agarose gel at 1.5%.

### IDS enzymatic assay and measurement of GAG content

Samples were obtained by homogenizing cells resuspended in NaCl 0.9%, through sonication by Sonics Vibra Cell (Sonics & Materials, Inc, Newtown, CT, USA). Debris was pelleted twice by centrifugation at 4 °C and supernatants were collected and assayed for total protein concentration (mg/ml) by the BCA assay (Thermofisher, Italy). Iduronate-2-sulfatase activity was determined by a two-step fluorimetric assay using the substrate 4-Methylumbelliferyl-a-L-Idopyranosiduronic Acid 2-sulfate (MU-a-ldoA-2S, Biosynth, Staad, Switzerland). Results were obtained using 4-methylumbelliferone as a standard and were normalized for total protein content. IDS activity was expressed as nmoles of MU-a-ldoA-2S substrate hydrolysed in 4 h per mg total proteins (nmol/4 h/mg). GAG content was measured as previously described^37^ and data were expressed as μg GAG per mg total proteins.

### Immunofluorescence staining

Twenty-five thousand cells per well were seeded on polyornithine/fibronectin-pretreated coverslips in 24-well plates and differentiated for five days. On the day of immunostaining differentiated cells were washed twice with PBS and fixed with 4% PFA for 20 min. After rinsing twice with PBS, coverslips were treated with methanol for 5 min at -20 °C, washed twice with PBT (PBS/0,05% Tween 20) and transferred to a humid chamber. A blocking step with 10% sheep serum for 1 h at room temperature was followed by an overnight incubation with a primary antibody at 4 °C. After three washes with PBT coverslips were incubated with secondary antibody at room temperature for 1 h and finally washed with PBT. All samples were mounted with Fluoromount (Thermofisher, Italy) after staining with 0.1 mg/ml concanavalin (Thermofisher, Italy) for 10 min or with 5,5 μM Hoescht (Thermofisher, Italy) for one hour to label whole cell membranes or nuclei, respectively. The list of primary antibodies is reported in Supplementary Table S2B.

### Fluorescent dye (Lysotracker, DCFDA, Nile Red and Top-Chol) staining

At day 5 of differentiation control and IDS mutant cells were labelled with 100 nM Lysotracker Red (Thermofisher, Italy) for 1 h at 37 °C. After two washes with PBS, cells were fixed with 4% PFA for 20 min and washed twice again with PBS. Concanavalin (0.1 mg/ml) counterstaining was performed for 10 min and after two brief washes with PBS, all coverslips were mounted in Fluoromount (Thermofisher, Italy). To detect ROS, we stained differentiated cells with H2-DCFDA (Molecular probes, Thermofisher, Italy) in HBSS (Hank Balanced Salt Solution) for 30 min at 37 °C. After brief washes with PBS and incubation with 5,5 mM Hoescht, cells were mounted and visualized under confocal microscopy. To verify lipids accumulation we stained differentiated cells at day 5 with 1 μM Nile Red (Merck, Italy) for 10 min at 37 °C and, after brief washes with PBS, we fixed stained cells for 20 min with 4% PFA. To visualize intracellular cholesterol, we first incubated differentiated cells with 0.5 mM TopFluor®Cholesterol (Top-Chol) (Merck, Italy) for 18 h, starting at day 4 of differentiation. At day 5 cells were washed with PBS and stained with Lysotracker Red for 1 h at 37 °C and post-fixed according to the abovementioned protocol.

### Western blot

All procedures were performed as previously described^38^. Briefly, differentiated cells at day 5 were harvested and lysed in Tissue Extraction Reagent (ThermoFisher, Italy) in the presence of protease and phosphatase inhibitors (ThermoFisher, Italy). Lysates were next centrifuged at 13,000 × g for 30 min at 4 °C. The supernatant was collected and protein concentration was determined by BCA Protein Assay Kit (ThermoFisher, Italy). Extracted proteins were supplemented with 4 × sample buffer (ThermoFisher, Italy), heated at 75 °C for 10 min and run onto precast SDS-PAGE (ThermoFisher, Italy). Proteins were transferred on PVDF membranes (Merk Millipore, Italy) in 25 mM Tris, 192 mM glycine, 20% methanol (v/v) and incubated with primary antibodies overnight at 4 °C. All primary antibodies were used at 1:1000 diluition. After three washes in TBST (Tris-buffered saline-0.1%Tween 20), an incubation with secondary horseradish peroxidase (HRP)-conjugated goat anti-rabbit and mouse IgG antibodies (Biorad, Milan, Italy) was performed at room temperature for 1 h at 1:2000 diluition. Chemiluminescent signals were detected incubating the blotted membranes with the Supersignal West Pico Chemioluminescent substrate kit (ThermoFisher, Italy), visualized by Image Quant Las 4000 (GE Healthcare, Milan,Italy) and analyzed by ImageJ software (https://imagej.nih.gov/ij/). A complete list of primary antibodies used for Western is reported in Supplementary Table S2B. Images provided for Western Blots of Figs. 2 and 3 are not full-length blots as the original blotted membranes have been cut according to the molecular weight bands, before antibody incubation. The original uncropped and uncontrasted images are reported in Supplementary Material.

### Mitotracker staining and electron microscopy

Differentiated cells (d5) were incubated with the MitoTracker™ Orange CMXRos dye (Thermofisher, Italy) for 45 min at 37 °C. After brief washes with PBS, cells were fixed with 4% PFA for 20 min at room temperature. Once completed a second round of washes with PBS, cells were incubated with 0.1 mg/ml concanavalin (Thermofisher, Italy) and finally mounted with Fluoromount medium (Thermofisher, Italy). For T.E.M. analyses differentiated cells at day 5 of differentiation, were fixed using 4% PFA dissolved in PBS for 1 h and incubated in 2.5% glutaraldehyde in 0.1 M sodium cacodylate buffer pH 7.4 ON at 4 °C. All samples were next post-fixed in 1% osmium tetroxide plus 1% potassium ferrocyanide in 0.1 M sodium cacodylate buffer for 1 h at 4 °C. After three washes with distilled water, samples were dehydrated in a graded ethanol series and embedded in an epoxy resin (Sigma-Aldrich). Ultrathin Sects. (60–70 nm) were obtained with an Ultratome Leica Ultracut EM UC7 ultramicrotome, counterstained with uranyl acetate and lead citrate and viewed with a Tecnai G2 (FEI) transmission electron microscope operating at 100 kV. Images were captured with a Veleta (Olympus Soft Imaging System) digital camera.

### Confocal microscopy imaging and data analyses

All fluorescent images were sequentially acquired using Leica Stellaris confocal microscope equipped with a charge-coupled device camera and processed with the Leica LASX software. Each cellular field was acquired with a Z-stack of 1 μm using a 63 × HC PL APO CS2 objective (NA = 1.4) or 40 × HC PL APO (NA = 1.3) and a 405, 495, 551 nm Laser at a 400 Hz scan speed. All images were processed with Fiji (https://imagej.net/software/fiji). For each sample six or seven fields were acquired for a total number of at least 200 cells per condition. The integrated density of the total sum of slices per field was used to compare the different conditions for each marker. The Pearson’s coefficient for the colocalization between Lysotracker/Lamp1 and Top-Chol positive spots was assessed by using the JaCoP plugin on acquired field images, while for all Mitotracker Red stainings images were processed using the MiNA plugin^39^. All statistical analyses were performed using GraphPad Prism 9. Paired and unpaired t-tests with Mann–Whitney correction were applied according to the analyzed data.

## Supplementary Information

Supplementary Information 1.

Supplementary Information 2.

Supplementary Information 3.

Supplementary Legends.

Supplementary Legends.

Supplementary Legends.

Supplementary Table 1.

Supplementary Table 2.

Supplementary Information 9.

Supplementary Information 10.

## Data Availability

All relevant data supporting the key findings of this study are reported within the article and its Supplementary Information. Additional information will be provided upon request to Authors.
