# Supplementary figures and images for: A novel CRISPR/Cas9-based iduronate-2-sulfatase (IDS) knockout human neuronal cell line reveals earliest pathological changes
Source: Sci Rep. 2023 Jun 25;13:10289. doi: 10.1038/s41598-023-37138-5 (PMC10290981; doi:10.1038/s41598-023-37138-5)

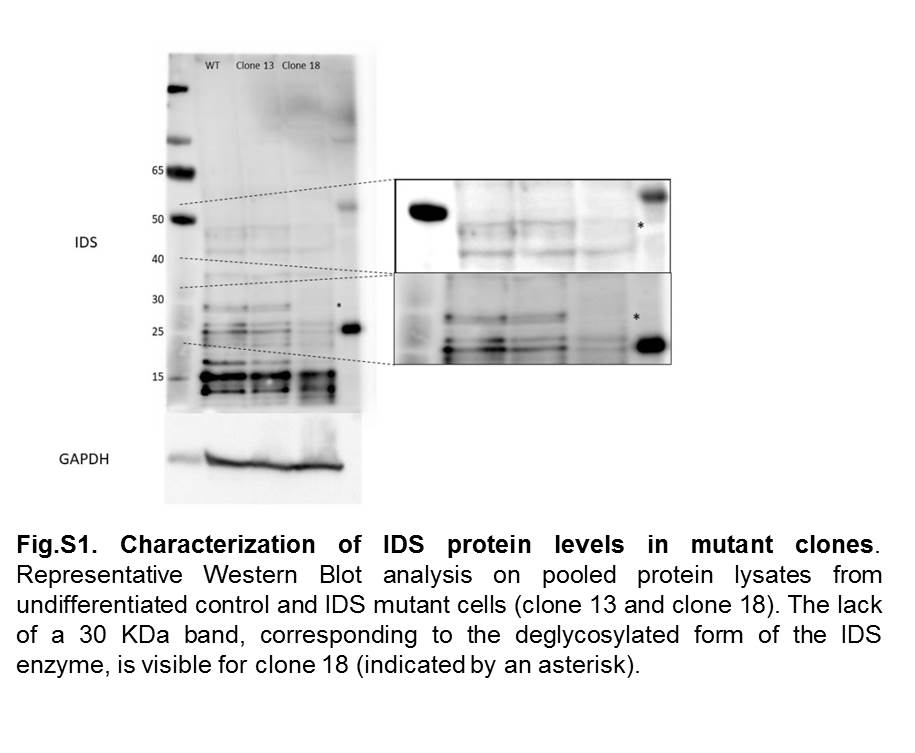

Supplement: Supplementary file 1 — Supplementary Information 1. [file 41598_2023_37138_MOESM1_ESM.jpg]

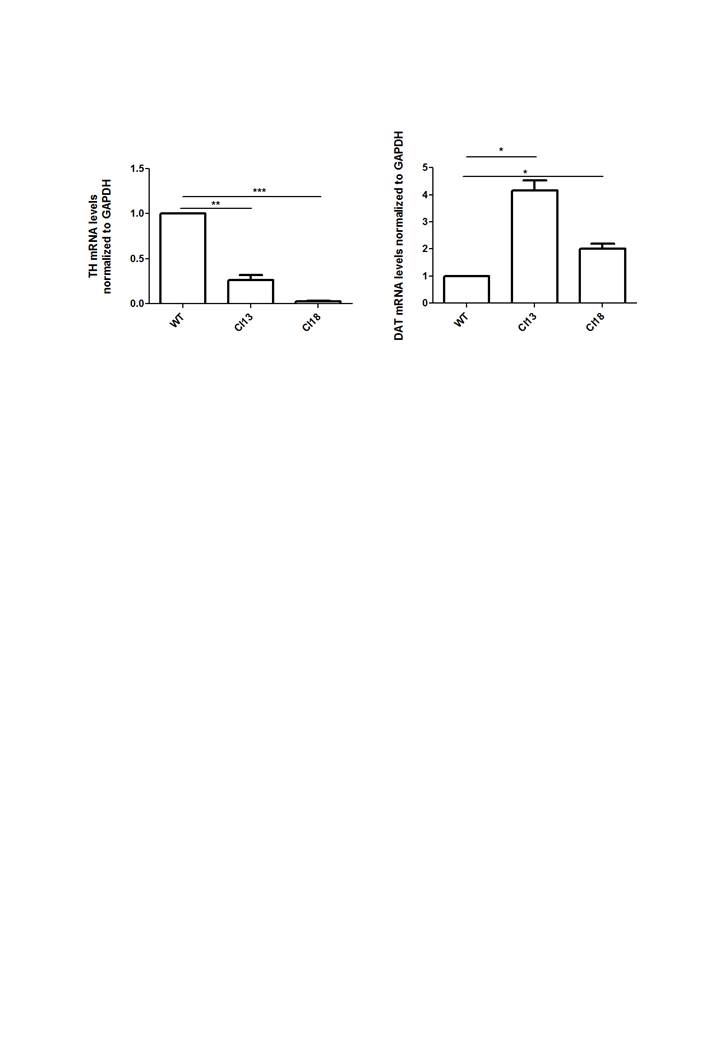

Supplement: Supplementary file 2 — Supplementary Information 2. [file 41598_2023_37138_MOESM2_ESM.jpg]

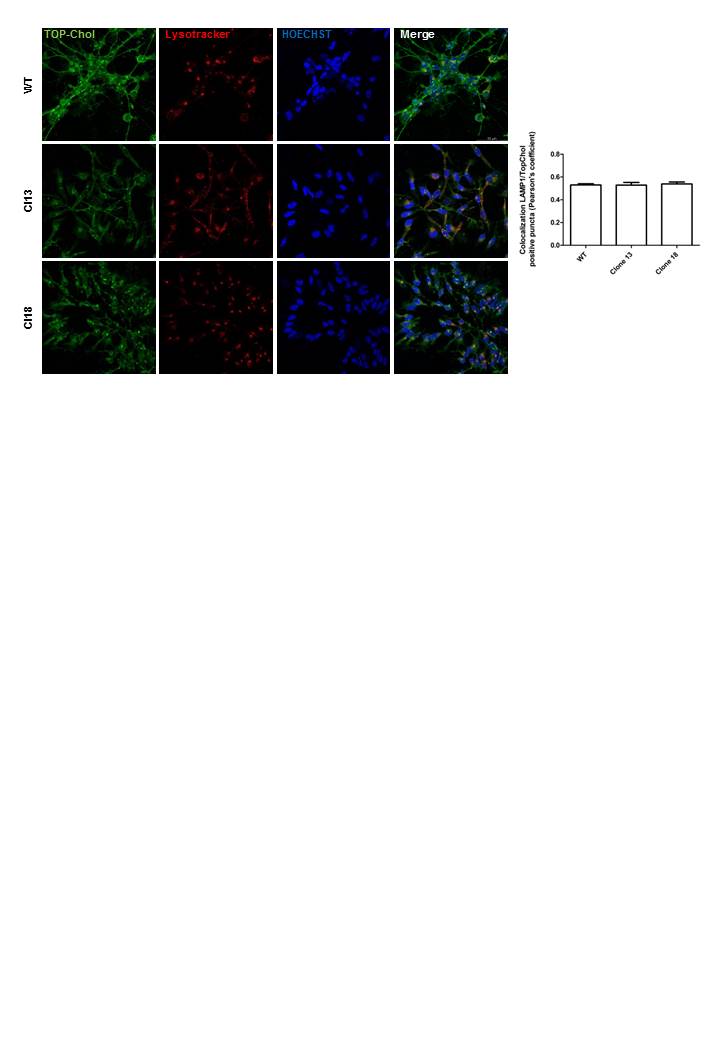

Supplement: Supplementary file 3 — Supplementary Information 3. [file 41598_2023_37138_MOESM3_ESM.jpg]

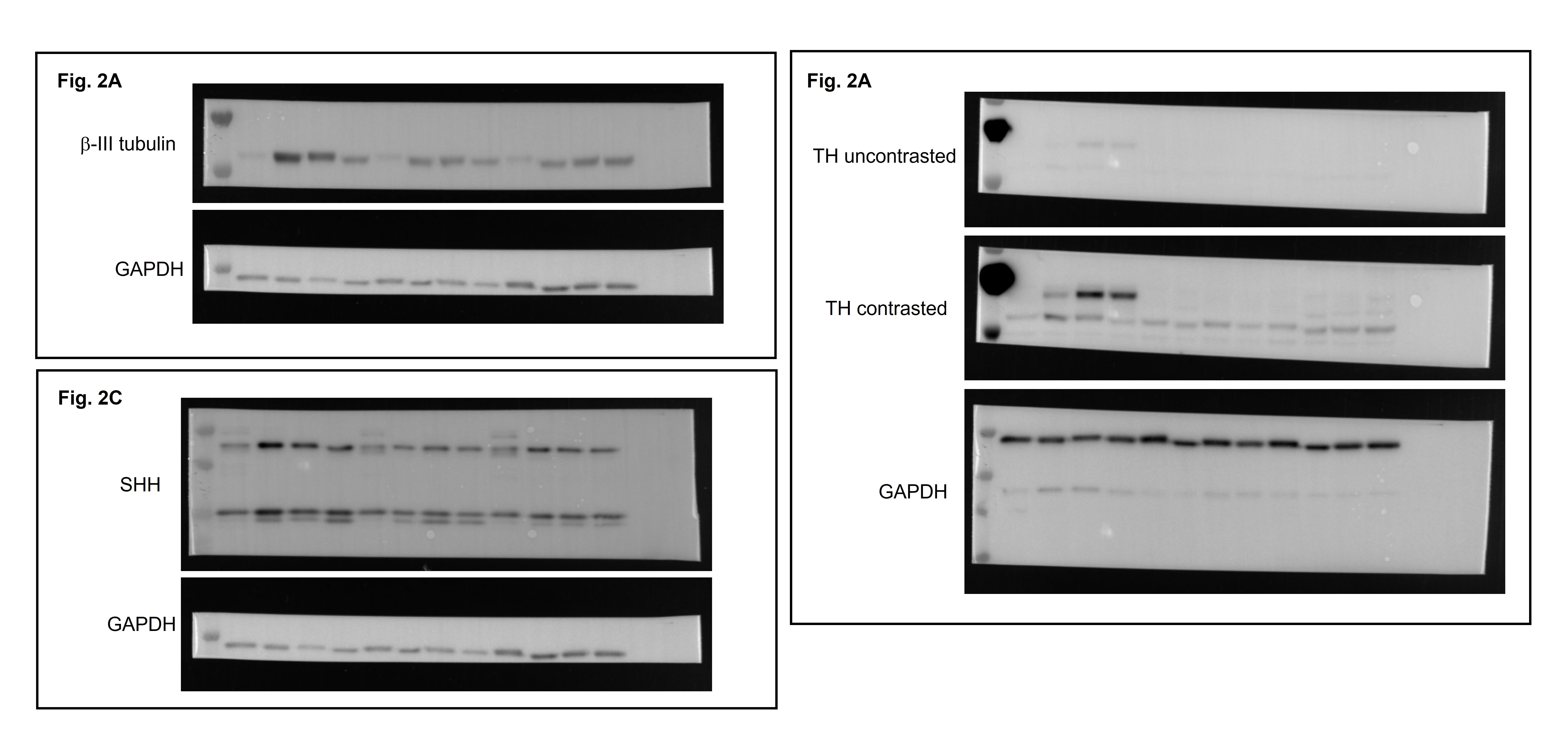

Supplement: Supplementary file 9 — Supplementary Information 9. [file 41598_2023_37138_MOESM9_ESM.jpg]

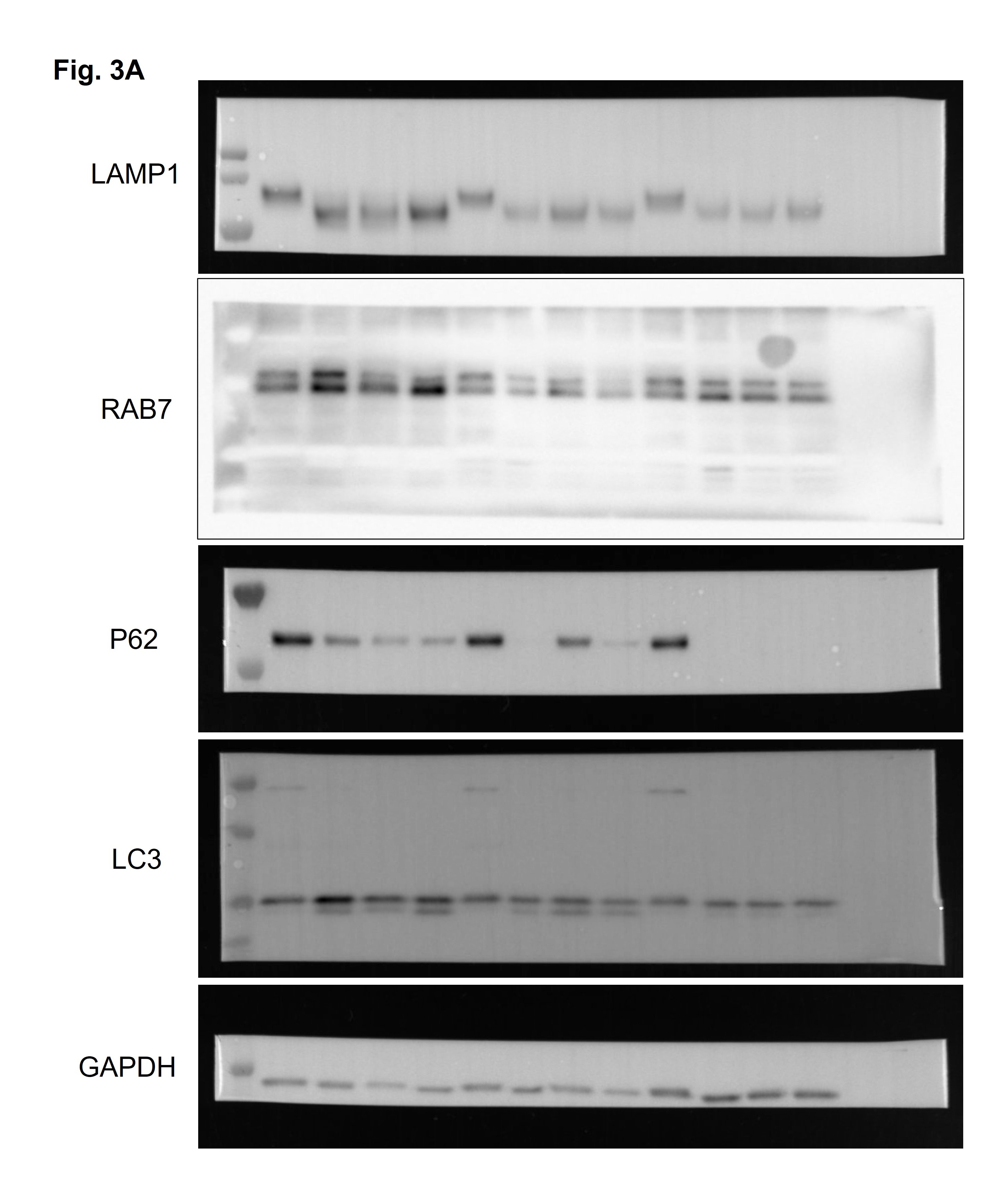

Supplement: Supplementary file 10 — Supplementary Information 10. [file 41598_2023_37138_MOESM10_ESM.jpg]
