# Supplementary material for: A novel CRISPR/Cas9-based iduronate-2-sulfatase (IDS) knockout human neuronal cell line reveals earliest pathological changes
Source: Sci Rep. 2023 Jun 25;13:10289. doi: 10.1038/s41598-023-37138-5 (PMC10290981; doi:10.1038/s41598-023-37138-5)
Supplement: Supplementary file 4 — Supplementary Legends. [file 41598_2023_37138_MOESM4_ESM.docx]

**Fig.S1.** **Characterization of IDS protein levels in mutant clones**. Representative Western Blot analysis on pooled protein lysates from undifferentiated control and IDS mutant cells (clone 13 and clone 18). The lack of a 30 KDa band, corresponding to the deglycosylated form of the IDS enzyme, is visible for clone 18 (indicated by an asterisk).
