# Supplementary material for: A novel CRISPR/Cas9-based iduronate-2-sulfatase (IDS) knockout human neuronal cell line reveals earliest pathological changes
Source: Sci Rep. 2023 Jun 25;13:10289. doi: 10.1038/s41598-023-37138-5 (PMC10290981; doi:10.1038/s41598-023-37138-5)
Supplement: Supplementary file 5 — Supplementary Legends. [file 41598_2023_37138_MOESM5_ESM.docx]

**Fig.S2. A reduced TH transcription is triggered by IDS loss of function.** Bar-graph showing TH and DAT1 mRNA levels assessed by RQ-PCR. Data are expressed as mean±SD of three independent replicates. (* p<0.05; **p<0.005; ***p<0.001; t-test).
