# Supplementary material for: A novel CRISPR/Cas9-based iduronate-2-sulfatase (IDS) knockout human neuronal cell line reveals earliest pathological changes
Source: Sci Rep. 2023 Jun 25;13:10289. doi: 10.1038/s41598-023-37138-5 (PMC10290981; doi:10.1038/s41598-023-37138-5)
Supplement: Supplementary file 6 — Supplementary Legends. [file 41598_2023_37138_MOESM6_ESM.docx]

**Fig.S3. No evident lysosomal cholesterol storage is detected in early differentiated MPS II neuronal cells.** Representative double TopChol/Lysotracker staining in differentiated (D5) control and mutated neurons. The bar graph on the right depicts the colocalization (Pearson’s coefficient) analyses performed on double-stained cells. Data are expressed as the mean±SD of four technical replicates of three independent biological replicates (400 cells were analyzed per condition of each biological replicate).
