# Supplementary material for: A novel CRISPR/Cas9-based iduronate-2-sulfatase (IDS) knockout human neuronal cell line reveals earliest pathological changes
Source: Sci Rep. 2023 Jun 25;13:10289. doi: 10.1038/s41598-023-37138-5 (PMC10290981; doi:10.1038/s41598-023-37138-5)
Supplement: Supplementary file 7 — Supplementary Table 1. [file 41598_2023_37138_MOESM7_ESM.docx]

| **Sequence** | **PAM** | **Score** | **#MM** | **Gene** | **Locus** |
| --- | --- | --- | --- | --- | --- |
| TATACGGAGAATCATCGGTA | TGG | N/A |  | IDS | chrX:+149501001 |
| TATCGGGAAAATCATCAGTA | AGG | 42 | 4 | Linc01256 | chr4:-132602335 |
| TATATGAAGAATTATAGGTA | TGG | 46 | 4 | Non-coding | chr15:-23461157 |
| TACAAGGAGAATAATCTGTA | AAG | 53 | 4 | Non-coding | chr5:+145566515 |
| TATATGAGAGAATCATCGTTA | TGG | 58 | 3 | Non-coding | chr4:-133200931 |
| TATAGAGAGAGTCATAGGTA | TAG | 58 | 4 | Non-coding | chr4:+129160180 |
| TATAAGGAAAATAATCAGTA | GGG | 61 | 4 | Non-coding | chr5:+30746710 |
| TATAAGGACAGTCATAGGTA | GAG | 66 | 4 | Non-coding | chr6:-144993964 |
| TATACTGTGAATTATAGGTA | CAG | 67 | 4 | Non-coding | chr18:+61098982 |
| TTTATGGAGAATCATCTGTG | CAG | 68 | 4 | Linc01256 | chr2:+2965729 |
| GACACGGAGACACATCGGTA | TGG | 69 | 4 | SMAD3 | chr15:+67181436 |
| TTTACAGAGAATCATTGTTA | GGG | 70 | 4 | SWAP70 | chr11:+9727539 |
| TATACTG-GAATCACCGGTA | CAG | 71 | 3 | ELF1 | chr13:+40948806 |
| AATATGGAGAATCAGAGGTA | CAG | 71 | 4 | ANKK1 | chr11:-113394278 |
| CATACAGAAAATCATTGGTA | GAG | 73 | 4 | DLGAP1 | chr18:-4352819 |
| TATACAGAGAA-CAACGGTA | AGG | 74 | 3 | Non-coding | chr10:+29166932 |
| TATATGAAGACTCAACGGTA | GAG | 74 | 4 | ZNF385D | chr3:-21933701 |
| TATAGAGAGATTTATCGGTA | GAG | 74 | 4 | Non-coding | chrX:+39595124 |
| TTTACAGAGAATCAGTGGTA | GGG | 80 | 4 | Non-coding | chr2:-133750543 |
| TATAAGGAGAATTATAGCTA | TGG | 83 | 4 | Non-coding | chr13:-69133840 |
| TACAAGGAGAATCATCTGTC | TGG | 83 | 4 | PSMB1 | chr6:-170536544 |
| TATACGAAGAATCATCATCA | GAG | 84 | 4 | Non-coding | chr13:-80482152 |
| AATACGGAGGATCAACTGTA | TAG | 84 | 4 | UBR1 | chr15:+43054018 |
| TATACGGAGAA-CATTGGTT | TGG | 87 | 3 | Non-coding | chr3:+184052194 |
| AAAACGGAGAATCATCTGGA | AGG | 87 | 4 | Non-coding | chr14:-38395480 |
| TCTACGGAAAATCTTCAGTA | AGG | 89 | 4 | SLC39A11 | chr17:+72868478 |
| TATAGGGAGAATGATCAGTG | GAG | 89 | 4 | Non-coding | chr4:-29826726 |
| TATACTGAGAACCATTGCTA | TAG | 91 | 4 | PTPRK | chr6:-128035709 |
| TAGACGGAGAACCTTCTGTA | AAG | 92 | 4 | IL10RB | chr21:-33294769 |
| TAGACGGAGAATCCTCCGTA | GAG | 93 | 3 | GAA | chr17:+80116490 |
| GAGACGGAGAATCAAGGGTA | AAG | 93 | 4 | NDUFAF2 | chr5:-61132344 |
| TCCACGGAGAATCAGCGGTC | TGG | 95 | 4 | SNRNP200 | chr2:+96282901 |
| TATATGGAGAATCTTTGATA | GAG | 95 | 4 | Non-coding | chr12:+39169413 |
| TATTCAGAAAATGATCGGTA | TAG | 96 | 4 | RAFGEF1A | chr10:+43247332 |
| TATACACAGAATCATCAGAA | AGG | 100 | 4 | Non-coding | chr2:-64213785 |
| TATAAGGAGAATGTTCAGTA | CAG | 100 | 4 | Non-coding | chr13:-26868561 |
| TAAACGGAAAACCATGGGTA | GGG | 103 | 4 | Non-coding | chr2:-24431566 |
| TATACGGAGTAACAATGGTA | GGG | 103 | 4 | CHL1-AS2 | chr3:+136391 |
| TATACAGAGAAACAACGATA | AGG | 103 | 4 | LINC01825 | chr2:+195539398 |
| TAAAAGGAGATTCATCGGTC | AGG | 104 | 4 | Non-coding | chr6:-136330718 |
| TATATGGAAAATTATGGGTA | TGG | 104 | 4 | Non-coding | chr6:-23901414 |
| TATACGAAGAATCATAGCTG | AGG | 104 | 4 | Non-coding | chr7:-145024879 |
| TATATGGAGAATCAACTCTA | GGG | 108 | 4 | GALNT10 | chr5:+154295633 |
| AATACTCAGAATCATGGGTA | CAG | 108 | 4 | Non-coding | chrX:+21016050 |
| CATACGGAGAAGCATCGGAT | GAG | 113 | 4 | TEAD4 | chr12:-3010087 |
| TATACAGATACTCATGGGTA | CAG | 116 | 4 | Non-coding | chrY:+15961334 |
| TATAGGGAGACTCAAGGGTA | GAG | 122 | 4 | Non-coding | chr13:-76796905 |
| TATACACAGAATCACCTGTA | GAG | 127 | 4 | RNLS | chr10:-88502694 |
| TATACAGAGAATCCACTGTA | GAG | 128 | 4 | Non-coding | chr2:-136062950 |
| TATACGGAGAAGAAGCGGTC | CAG | 143 | 4 | WDFY2 | chr13:+51596968 |
| TATCCGGAGAATGATGGGGA | AAG | 144 | 4 | Non-coding | chr9:-127986638 |
| TATACGGAGAAACAACTGAA | GAG | 147 | 4 | Non-coding | chrX:-33772468 |
| TATACGCAGAATCTTTGCTA | AAG | 149 | 4 | Non-coding | chr16:+8274956 |

**Table S1. List of predicted off-target regions for the designed *IDS* sgRNA .** The indicated scores denote very low potential editing, except for the IDS genomic region.
