# Supplementary material for: A novel CRISPR/Cas9-based iduronate-2-sulfatase (IDS) knockout human neuronal cell line reveals earliest pathological changes
Source: Sci Rep. 2023 Jun 25;13:10289. doi: 10.1038/s41598-023-37138-5 (PMC10290981; doi:10.1038/s41598-023-37138-5)
Supplement: Supplementary file 8 — Supplementary Table 2. [file 41598_2023_37138_MOESM8_ESM.docx]

**Table S2a.** Sequences of the sgRNA and primers used for genotyping and RT-PCR

| **sgRNA sequence** | **5’ –TACCGATGATTCTCCGTATA-3’** |  |
| --- | --- | --- |
| **Ids1for** | **-5’-GGTTCCACTTGCCCATTTGT-3’** | **T_annealing_: 60°C** |
| **Ids1rev** | **-5’ GGGAAGGGTGAGGATTTGGA-3’** |  |
| **Ids internalfor** | **5’- CCTCTGCTGCCTCCTTCATA-3’** | **T_annealing_: 63°C** |
| **Ids internal rev** | **5’- TGCATGTTCTGTGAAGCTGG-3’** |  |
| **IDS exon1 for** | **5’- AGTCTTCATGGGTTCCCGAC-3’** | **T_annealing_: 60°C** |
| **IDS exon 1 rev** | **5’- GAGCTCAGAACCAGACCCAG-3’** |  |
| **IDS exon2-5 for** | **5’-CACAGCCTCCTCTTCCAGAA-3’** | **T_annealing_: 60°C** |
| **IDS exon2-5 rev** | **5’-GCTTATGATACCCAACGGCC-3’** |  |

**Table S2b.** List of antibodies used for Western Blots and immunofluorescence

| **Antibody** | **Host** | **Distributor** |
| --- | --- | --- |
| **Acetylated tubulin** | Mouse | Merck T7451 |
| **Tyrosine Hydroxylase (TH)** | Rabbit | Merck-Millipore AB152 |
| **β-Tubulin III (neuronal)** | Mouse | Merck T8578 |
| **LAMP1** | Mouse | Thermofisher 14-1079-80 |
| **RAB7** | Mouse | Abcam 50533 |
| **LC3I/LC3II** | Rabbit | Thermofisher PA1-16930 |
| **p62** | Rabbit | Cell Signaling #5114 |
| **GAPDH** | Rabbit | Abcam AB9485 |
| **SHH** | Rabbit | Abcam AB19897 |
| **IDS** | Mouse | Abcam ab70025 |
